# Analysis of the Consolidation Phase of Immunological Memory within the IgG Response to a B Cell Epitope Displayed on a Filamentous Bacteriophage

**DOI:** 10.3390/microorganisms8040564

**Published:** 2020-04-14

**Authors:** Francesca Mantile, Angelo Capasso, Piergiuseppe De Berardinis, Antonella Prisco

**Affiliations:** 1 Institute of Genetics and Biophysics, CNR, 80131 Naples, Italy; francesca.mantile@igb.cnr.it (F.M.); angelocapasso27@libero.it (A.C.); 2 IBBC, CNR, 80131 Naples, Italy

**Keywords:** bacteriophage, phage-display, B cell epitope, β -amyloid, immunological memory, IgG, secondary response

## Abstract

Immunological memory can be defined as the ability to mount a response of greater magnitude and with faster kinetics upon re-encounter of the same antigen. We have previously reported that a booster dose of a protein antigen given 15 days after the first dose interferes with the development of memory, i.e., with the ability to mount an epitope-specific IgG response of greater magnitude upon re-encounter of the same antigen. We named the time-window during which memory is vulnerable to disruption a “consolidation phase in immunological memory”, by analogy with the memory consolidation processes that occur in the nervous system to stabilize memory traces. In this study, we set out to establish if a similar memory consolidation phase occurs in the IgG response to a B cell epitope displayed on a filamentous bacteriophage. To this end, we have analyzed the time-course of anti-β-amyloid IgG titers in mice immunized with prototype Alzheimer’s Disease vaccine fdAD(2-6), which consists of a fd phage that displays the B epitope AEFRH of β -amyloid at the N-terminus of the Major Capsid Protein. A booster dose of phage fdAD(2-6) given 15 days after priming significantly reduced the ratio between the magnitude of the secondary and primary IgG response to β-amyloid. This analysis confirms, in a phage vaccine, a consolidation phase in immunological memory, occurring two weeks after priming.

## 1. Introduction

Long-lasting antibody responses and immunological memory are the desired outcomes of vaccination [1,2,3]. Immunological memory, i.e., the ability to mount an enhanced response to an antigen that has been previously encountered, is a system-level property of the immune system, that arises from an increase in the frequency of antigen-specific B and T lymphocytes, as well as from the differentiation of antigen-experienced lymphocytes into qualitatively different cell populations, namely memory cells, which display faster response to antigen re-exposure and the ability to self-renew [4,5,6,7,8].

We have previously observed, in a dataset of one-year time-courses of IgG titers against a B cell epitope of β-amyloid (Aβ) in BalbC mice immunized with prototype Alzheimer’s Disease vaccine (1-11)E2, a multimeric protein, in which the magnitude of the IgG responses upon late antigen re-encounter is not always enhanced compared to the primary response [9,10]. Mice immunized with a single dose of (1-11)E2, when immunized with a recall dose nine months later, display a second peak of anti-β-amyloid IgG that can be higher, of the same height, or lower than the first peak. Memory, defined as the acquired ability to display an enhanced response, and operationally defined in our analysis of IgG titer trajectories as a ratio between the height of the secondary and primary IgG peaks above two, only occurred in a subset of individuals. In the same dataset, we observed a statistically significant decrease in the ratio between the height of the secondary and primary response IgG peaks in a treatment group that had received a booster injection of antigen 15 days after the first dose. Treatment groups that had received the booster injection 7 or 21 days after the first dose displayed no statistically significant “memory” differences from the single-dose group. We interpreted these observations hypothesizing that, when a booster dose is given 15 days after the first dose, it disrupts the development of immunological memory. By analogy with the memory consolidation processes that occur in the nervous system to stabilize memory traces, we named the time window during which the development of immunological memory is vulnerable to a disrupting stimulus “a consolidation phase of immunological memory [10]”.

The concept of a consolidation phase of immunological memory still awaits the identification of its cellular correlates and confirmation in other immunization protocols. It is possible that the memory consolidation phase only occurs in immunizations with antigens characterized by specific structural features or by quantitative or qualitative features of the B or T cell responses. In the present study, we set out to establish if the consolidation phase in immunological memory is confirmed in a different IgG titers dataset, namely in the IgG response to B cell epitope 2-6 of Aβ after immunization with vaccine fdAD(2-6), a prototype vaccine for the prevention of Alzheimer’s Disease consisting of a filamentous bacteriophage [11,12].

Vaccine fdAD(2-6) consists of filamentous phage fd, engineered to express two forms of the Major Capsid Protein pVIII, namely the wild type pVIII and a recombinant pVIII, that displays the epitope AERFH of Aβ on the phage surface [11]. As the first two amino acids of the processed N-terminus of the pVIII protein are an alanine (A) and a glutamic acid (E), only three extra amino acids were inserted in the recombinant pVIII, namely sequence FRH, to obtain sequence AEFRH [11]. Each fdAD(2-6) phage particle displays, on average, 800 copies of the AEFRH epitope [11]. Monthly immunizations with fdAD(2-6) afford a significant reduction of Aβ plaque pathology in a mouse model of Alzheimer’s Disease [11]. Phage vaccine fdAD(2-6) differs from the (1-11)E2 vaccine that was initially used to identify the memory consolidation phase in the nature of the vaccine carrier (filamentous phage versus multimeric protein), in the polarization of the T cell response (TH1 versus TH2), and in the length of the B cell epitope (5 aminoacid Aβ peptide AEFRH versus 11 aminoacid Aβ peptide DAEFRHDSGYE) [9,10,11,12,13,14].

We report here that, in mice immunized with fdAD(2-6), a booster dose at day 15 significantly reduced the ratio between the magnitude of the secondary and primary response peaks, reproducing the phenomenon we had previously observed in the immunization with a multimeric protein^13^. The results of this study are in good agreement with the consolidation phase model of immunological memory [10], which proposes that there is a time window during which the formation of immunological memory is vulnerable, and a booster dose acts as a disrupting stimulus.

## 2. Materials and Methods

### 2.1. Mice

All experiments were performed on female BalbC mice, purchased from Charles River Laboratory, Italy. The first dose of vaccine was injected when the mice were 8 weeks old. Protocols involving mice were carried out in accordance with European Union Laws and guidelines (European Directive 2010/63/EU) and under the authorization 161/2015-PR released by the Italian Ministry of Health.

### 2.2. Model Vaccine

The vaccine fdAD(2-6) is a filamentous bacteriophage fd, displaying epitope 2–6 (AEFRH) of Aβ at the N-terminus of Major Capsid Protein [11]. The concentration of purified phages was measured assuming that a 1 mg/mL phage solution gives a reading of 3.8 o.d. at 269 nm. The ratio of recombinant pVIII to wild type pVIII was calculated by performing N-terminal sequencing of phages and calculating the ratio of the yield of amino acids deriving from the recombinant and wild type pVIII. Each vaccine dose consisted of 6 µg of epitope 2-6 displayed on fdAD(2-6), mixed with 100 μL of Freund’s adjuvant, in a final volume of 200 μL. Complete Freund’s Adjuvant (CFA) was used in the first injection, and Incomplete Freund’s Adjuvant (IFA) was used in subsequent shots.

### 2.3. Immunization and Bleeding Schedules

The vaccine was injected intraperitoneally. We have monitored, for a total of 12 months, the time course of the antibody response in mice undergoing 4 different dosing schedules, as previously described [10]. All dosing schedules included a first dose given when the mice were 2 months old and a recall dose given 9 months after the first dose (day 274). One group received only the first dose and the recall dose, while the other groups also received a booster dose, respectively 1, 2, or 3 weeks after the first dose.

Blood was collected from the tip of the tail, with heparinized microhematocrit capillaries, at the following time points after the first dose: day 14, 35, 42, 88, 273, 288, 302, 323, and 361. Blood was left at room temperature for 30 min, then centrifugated at 6000 rpm for 30 min. The serum was divided into aliquots and stored at −80 °C.

### 2.4. Antibody Titer Measures

The anti-Aβ and anti-fd IgG antibody titers were measured by ELISA assays, as previously described [9,11].

### 2.5. Statistical Analysis and Data Availability

The Kolmogorov–Smirnov test was used to test for the normality of data distribution. Data with a log-normal distribution were log-transformed prior to statistical analysis. The significance of differences between each group of boosted mice (D7B, D15b, and D21B) and the SD group was evaluated with the unpaired t-test. Correlations were analyzed with Pearson Correlation Coefficient. The Mann Whitney test for unpaired data was used to compare data when a normal distribution could not be demonstrated. All data generated or analyzed during this study are included in this article.

## 3. Results

### 3.1. Generation of a Dataset of Antibody Titers Aimed at Testing the Model of a Consolidation Phase of Immunological Memory in the Context of the IgG Response to a Phage-Displayed B Cell Epitope

A consolidation phase in immunological memory is defined operationally as the time window during which a booster dose reduces the subsequent immunological memory, that is the ability to display an enhanced response to a late re-encounter [10]. The consolidation phase in immunological memory was first identified in the IgG response to a B cell epitope of β-amyloid displayed on a multimeric protein [10]. We report here a new dataset of antibody titers, with the aim to establish if a consolidation phase of immunological memory can be identified in the IgG response to a phage-displayed B cell epitope; the immunization and bleeding schedule is designed to allow the identification of the time window during which memory consolidation occurs.

**Figure 1:**
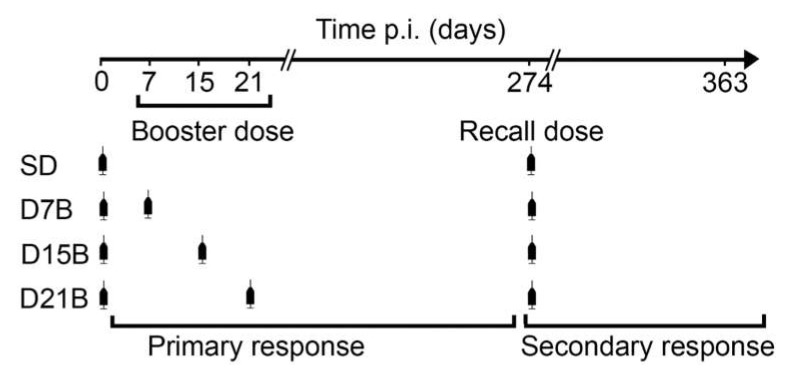
Experimental design for the identification of a consolidation phase in immunological memory (Figure 1 in [10]). The single-dose (SD) control group received a single dose of vaccine fdAD(2-6). Other treatment groups also received a booster dose, respectively, at day 7 (D7B), day 15 (D15B), and day 21 (D21B) after the first dose. All mice received a recall dose 9 months after the first dose and were monitored for 3 more months. Primary response is defined as the response initiated by the first dose of vaccine (days 0–274 post immunization), secondary response is defined as the response initiated by the recall dose (days 274–363 post immunization). In all groups *n* = 10.

Figure 1 schematizes the immunization protocol utilized, the 4 treatment groups, and the definitions of booster dose, recall dose, primary response and secondary response utilized throughout this study.

The single-dose (SD) group is the control group of our analysis, as it exemplifies the IgG kinetics of the primary and secondary response when the immune response initiated by the first dose is not perturbed by early booster doses. The other treatment groups received a booster dose of antigen at an early timepoint after the first dose, namely 7, 15, or 21 days after the first dose, with the aim to establish if, at these timepoints, antigen re-encounter impairs the development of immunological memory. The sera were collected at timepoints (day 14, 35, 42, 88, 273, 288, 302, 323, and 361) identified in preliminary experiments as sufficient to capture the trajectory of the primary and secondary IgG responses [10].

**Figure 2:**
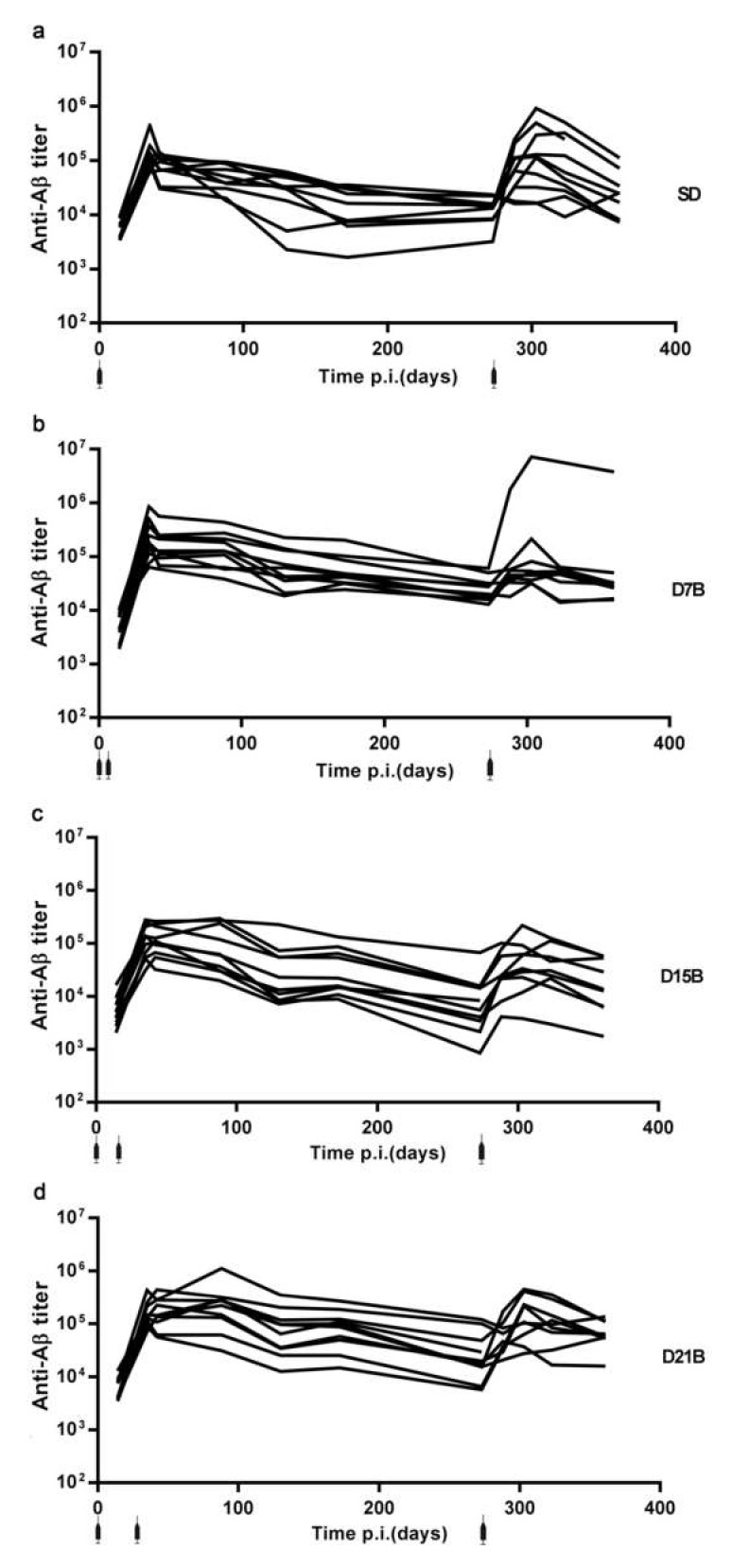
Time course of the IgG titer against Aβ. The line graphs show the trajectory of the IgG titer in each mouse (*n* = 10 per group): (**a**) Control group SD, (**b**) treatment group D7B, (**c**) treatment group D15B, (**d**) treatment group D21B. Injections are shown. Serum was sampled at 9 timepoints: day 14, 35, 42, 88, 273, 288, 302, 323, and 361.

Figure 2 reports the entire anti-β-amyloid IgG dataset, showing the time-courses of anti-β-amyloid IgG titers in mice immunized with vaccine fdAD(2-6) with the four different dosing protocols.

All 40 mice immunized with fdAD(2-6) mounted an antibody response to Aβ (Figure 2), confirming that fdAD(2-6) is an effective immunogen for the induction of an antibody response to β-amyloid.

#### 3.1.1. Analysis of the Consolidation Phase of Immunological Memory in the IgG Response to a Phage-Displayed B Cell Epitope

The “consolidation phase of immunological memory” hypothesis poses that a booster dose of antigen, if given during the consolidation phase, which is a time-window observed around two weeks following the first exposure to antigen, impairs the ability to subsequently display an enhanced response to a recall dose [10]. To analyze the enhancement of the IgG titer between the primary response and the secondary IgG responses, we computed the ratio between the peaks of the secondary and primary IgG responses to Aβ in the dataset shown in Figure 2a–d. We define the peak of the primary response to be the higher value of IgG titer between day 0 and day 273 and the peak of the secondary response to be the higher value of IgG titer after day 273.

**Figure 3:**
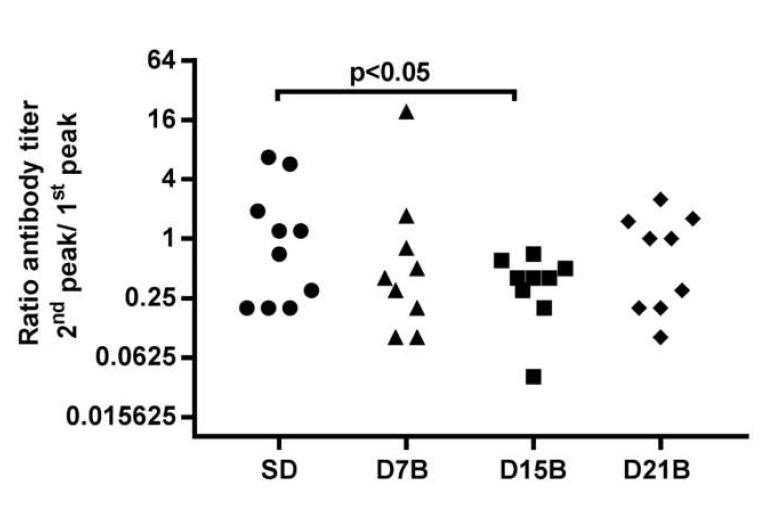
The dot plot shows the ratio between the magnitude of the peak of the secondary and primary anti-Aβ IgG response in individual mice from groups SD, D7B, D15B, and D21B. In this analysis, a high ratio indicates that the individual mounted a secondary response of greater magnitude than the primary response, which is the operative definition of immunological recall memory. In the D15B group that received a booster dose at day 15, the ratio between the magnitude of the peak of the secondary and primary IgG is significantly reduced (*p* < 0.05, t test) compared to the control group, indicating that the day 15 dose impaired the development of immunological memory.

The ratio between the peak of the secondary and primary anti-Aβ IgG response ranged from 0.2 to 7 in the control group SD that did not receive a booster dose (Figure 3). The ratio between the magnitude of the secondary and primary response was significantly lower (*p* < 0.05) in the D15B treatment group, compared to the control SD group. On the other hand, in the D7B and D21B group, the ratio between the magnitude of the secondary and primary response was not significantly different from the ratio observed in the SD control (Figure 3). So, this analysis is in good agreement with the hypothesis that there is consolidation phase of immunological memory in the IgG response to B cell epitope AERRH of Aβ displayed on phage-based vaccine fdAD(2-6). Overall, the timing of the consolidation phase window appears the same as previously described in the case of a multimeric protein antigen [10].

**Figure 4:**
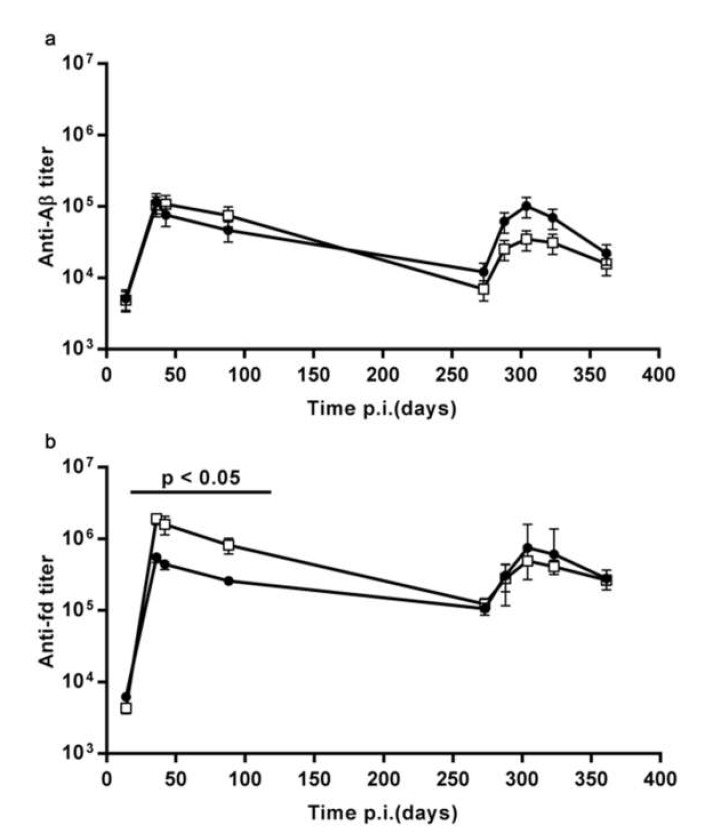
The graphs report the time course of the IgG titer against Aβ (**a**) and fd (**b**), GMT ± SEM, of the SD group (full circles) overlayed to the D15B group (open squares). In both groups *n* = 10. The primary IgG response against Aβ and fd is not reduced by a day 15 booster dose.

To rule out an alternative explanation, we asked if the booster dose at day 15 had reduced the primary response. During the primary response, there was no statistically significant difference in the anti-Aβ titer between the mice in the SD and D15B treatment groups (Figure 4a). The anti-fd titer was increased at early time points (14 to 88 days) in mice that had received a day 15 boost, but at the time of recall the anti-fd titer was the same in the SD and D15B groups (Figure 4b). We conclude that the booster dose of fdAD(2-6) at day 15 impaired immunological memory to Aβ without impairing the primary response to Aβ and to fd.

#### 3.1.2. The Pre-Existing Antibody Titers Against Aβ and Against the fd Phage do not Affect the Ability to Display an Enhanced Response to Recall

Mice immunized with fdAD(2-6) developed an antibody titer both to the Aβ(2-6) peptide and to the fd phage. In all mice, the titers were still measurable when the recall dose was injected, nine months after the first dose. We asked whether, at the time of recall, the pre-existing antibody titer against Aβ or against the fd phage compromised the secondary response against Aβ. To this end, we have calculated the coefficient of correlation between the IgG titer at the time of recall, i.e., at day 274, and the secondary/primary anti-Aβ IgG peak titer ratio. Neither the anti-Aβ, nor the anti-fd IgG titer at recall correlate with the secondary/primary anti-Aβ IgG peak titer ratio; Pearson’s correlation coefficients are 0.16 and 0.10 respectively. Therefore, in this setting, the pre-existing antibody titers against Aβ and against the fd phage do not affect the affect the ability to display an enhanced response to recall.

**Figure 5:**
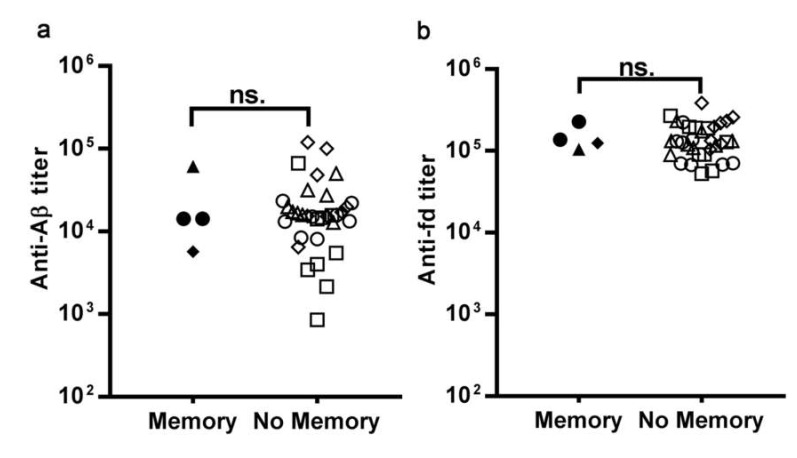
The dot plots show the IgG titer against Aβ (**a**) and fd (**b**) at day 273, the day before the recall dose, in mice that displayed an enhanced response (peak magnitude ratio over 2- Memory) and all other mice (peak magnitude ratio below or equal to 2- No Memory). Each dot represents a mouse of the control SD group (circles), D7B group (triangles), D15B group (squares), D21B group (diamonds). There is no statistically significant difference (*p* = 0.43, Mann Whitney test) in the pre-existing antibody titer against Aβ and fd at the time of recall between mice that did or did not display enhanced secondary responses (i.e., memory) to Aβ.

We also specifically asked if the mice that exhibited immunological memory to Aβ, i.e., a secondary response of enhanced magnitude, differed from the other mice with respect to their pre-existing anti-Aβ and anti-fd titer at recall. As previously described [10], we classified as “immunological memory” the responses with a peak of the secondary IgG response to Aβ more than two-fold higher than the peak of the primary response. Only 4/40 mice in this dataset displayed immunological memory; we compared the anti-Aβ and anti-fd IgG titers at recall of the four mice that had displayed memory, with the titers of the other, no-memory mice (Figure 5). We observed no significant difference in the anti-Aβ IgG titer (Figure 5a) and the anti-fd IgG titer (Figure 5b) at the time of recall between the mice that displayed a memory response to Aβ and the mice that did not. Therefore, in this experimental setting, pre-existing antibodies against Aβ or against the carrier fd do not affect the ability to display an enhanced response to recall.

## 4. Discussion

In this study, we tested, in the context of a phage-based vaccine, the model that postulates a consolidation phase in immunological memory, that states that there is a time window, after the first encounter with an antigen, when immunological memory can be disrupted by a second dose. To this end, we analyzed the trajectories of the IgG titer against β-amyloid in inbred mice immunized with prototype vaccine fdAD(2-6), that consists of a filamentous phage fd displaying nearly 800 copies of a B cell epitope of β-amyloid. When we calculated the ratio between the magnitude of the peak of the secondary and primary response to β-amyloid in individual mice, we observed a continuum; the recall response of a mouse could be higher, equal, or lower than the primary response of the same individual, as evidenced by a ratio ranging from 0.2 to 7 in the control single-dose group. A booster dose at day 15 significantly shifted this continuum toward lower values, whereas booster doses at day 7 or 21 had no statistically significant effect.

We have previously reported that vaccine fdAD(2-6) induces an antibody response to the β-amyloid in mice even when no adjuvant is used [11]. In this study, the CFA-IFA adjuvants were used, so as to replicate the experimental protocol first used to identify the consolidation phase [10] without adding variables other that the antigen-delivery system used. The analysis of the antibody titer trajectories performed in this study requires the primary response to the epitope to be detectable in all individuals after a single dose of vaccine, hence the need for a robust immunization protocol.

The results of this study support the concept that there is a consolidation phase in immunological memory, namely a time window during which the formation of memory is vulnerable, and an additional dose acts as a disrupting stimulus. We had previously identified a consolidation phase in immunological memory to the B epitope (1-11) of Aβ displayed on the multimeric protein (1-11)E2, an antigen that induces a TH2-skewed response; here we report a consolidation phase in immunological memory to the B epitope (2-6) of Aβ displayed on the filamentous bacteriophage fd, an antigen that induces a TH1-skewed response. Both vaccine (1-11)E2 and vaccine fdAD(2-6) are highly repetitive antigens and, in both studies, CFA-IFA adjuvants were used. It is possible that different types of antigen, adjuvants, or injection routes, and different doses may be associated with differences in the kinetics of the response. Interestingly, we observed the same timing of the consolidation phase in the case of the phage vaccine analyzed in this study and the multimeric protein vaccine analyzed previously.

Booster-induced hyporesponsiveness has been previously observed in polysaccharide vaccines. Unconjugated meningococcal polysaccharide vaccination induces antibody hyporesponsiveness, that impairs antibody responses to subsequent injections of meningococcal polysaccharide (MPS) or meningococcal conjugate vaccines. Administering MPS as a probe to assess conjugate vaccine-induced immunologic memory also can extinguish subsequent memory anticapsular antibody responses, whereas conjugate vaccination regenerates memory B cells [15]. It has been hypothesized that the polysaccharide, a T independent antigen, may stimulate the existing pool of memory B cells to differentiate into plasma cells and secrete antibody without replenishment of the memory B cell pool [15]. A study on the effect of 1, 2, or 3 boosters of pneumococcal polysaccharide with 16 day intervals in mice primed with a pneumococcal conjugate concluded that booster-induced hyporesponsiveness is caused by abrogation of conjugate-induced GC reaction and depletion of polysaccharide-specific antibody-secreting cells, resulting in no homing of new specific long-lived plasma cells to the bone marrow [16]. Different from our study, the pneumococcal polysaccharide booster reduced the antibody titer of boosted mice, compared to the PBS control; instead, we did not observe a titer reduction, indicating that in our experimental system the booster dose affected the development of memory but did not reduce the number of antibody secreting cells; a difference in the study design is the age of mice at the time of priming: seven days in the study on pneumococcal polysaccharide and eight weeks in the present study.

This study revealed a previously unappreciated degree of interindividual variability in the development of immunological memory to Aβ in BalbC mice immunized with phage-based vaccine fdAD(2-6). Interindividual variability may prove a particularly serious problem for vaccines that focus the antibody response on a precise target, for instance, a short B cell epitope, as in the case of many second generation anti-Aβ vaccines for the prevention of Alzheimer’s Disease. Since a clinical trial of immunization of Alzheimer’s Disease patients with the full-length Aβ peptide 1-42 caused aseptic meningoencephalitis in some individuals [17], with evidence of a T cell infiltrate in the brain, several anti-Aβ vaccines have been generated that do not contain T cell epitopes of β-amyloid and combine a defined B cell epitope with a carrier [18,19,20,21,22,23,24].

Filamentous bacteriophage fd has been proposed as an antigen delivery system for vaccination against tumors, parasites, viruses, and neurodegenerative diseases [11,12,25,26,27]. Our results suggest that the design of B epitope-specific immunization protocols based on phage fd should take into account the interindividual variability in the strength of the secondary IgG response and the possible disruptive effect of a day 15 booster dose on immunological memory. Bacteriophages have also been safely used as sensor of specific antibody responses in patients with immunodeficiencies [28]; in this study, we have used the fd bacteriophage as a model antigen to study the humoral immune response, and in particular, to confirm the model of a consolidation phase in immunological memory, a model originally derived from observations with a multimeric protein antigen [10]. The multimeric protein antigen induced an enhanced secondary response in the majority of immunized mice [10], and therefore, being an efficient inducer of memory, it is a good model antigen to analyze interventions that disrupt memory; the filamentous bacteriophage, on the other hand, under the same immunizing conditions induced an enhanced secondary response only in a minority of immunized mice, and therefore the bacteriophage is a promising model antigen to test interventions that enhance the development of recall memory.

Early boosters are not usually included in immunization protocols [29], so the relevance of the identification of a consolidation phase in immunological memory does not lie primarily in the optimization of immunization protocols; rather, the results of our studies pave the way to the design of cellular immunology and systems immunology experiments aimed at understanding, at a quantitative level, how developmental processes in the early phases of the immune response affect the magnitude of subsequent responses, i.e., immunological memory. It is possible to speculate that, in this experimental system, the booster dose interferes with a different stage of the Germinal Center (GC) reaction, depending on its precise timing. Following a booster immunization, new B cell clones can populate pre-existing GC [30,31]. Antigen is supposed to be one of the main limiting factors for the entrance of B cells into GCs [30,31]. The output of the GC response undergoes a temporal switch [32]. Unswitched memory B cells are generated early in the response, followed by switched memory B cells, and finally by a delayed appearance of isotype-switched bone marrow long-lived plasma cells [32]. In this experimental system, we never observed, in prime-boosted mice, a reduced primary response compared to single dose mice, indicating that the booster doses did not inhibit the development of antibody secreting cells. Memory cells are characterized by their self-renewal ability; it is possible to speculate that, at day 15, the developing memory cells have not fully acquired yet their ability to self-renew, and therefore, if a second dose activates their proliferation, the number of memory cells decreases.

## 5. Conclusions

We report here that a booster dose of a filamentous bacteriophage vaccine, injected 15 days after the primary immunization, impaired the antibody response to a recall dose, administered nine months later. This finding confirms, in the context of immunization with a phage, the occurrence of a consolidation phase in immunological memory [10]—that is a time window during which the formation of immunological memory is vulnerable, and a booster dose acts as a disrupting stimulus.
